# Special Issue “Women in Science”—The First Edition

**DOI:** 10.3390/bios13040438

**Published:** 2023-03-30

**Authors:** Cecilia Cristea

**Affiliations:** Department of Analytical Chemistry, Faculty of Pharmacy, “Iuliu Hatieganu” University of Medicine and Pharmacy, 4 Pasteur Street, 400349 Cluj-Napoca, Romania; ccristea@umfcluj.ro; Tel.: +40-264-597-256

This Special Issue entitled “Women in Biosensors” has been launched to celebrate and highlight the achievements of women in the biosensors research area, presenting biosensor-related work performed in groups leaded by women scientists. The main goal of this Special Issue was to further encourage and promote the scientific contributions of women researchers in this field. Another goal was to encourage young researchers to choose this field of research in the realm of bioelectrochemistry.

It is not an easy task to be a female scientist. Women are also mothers, helpers, leaders, scientists, and, just like men, women are trying to improve the quality of life and to make a difference in this world by addressing current global challenges. However, when working in an academic world, it is easy to notice that female scientists often put their career on hold if they decide to have family and children; furthermore, a significant amount of effort and energy is invested into recovering the time spent away from the lab.

It is important to note that female scientists can bring important contributions to society by investing their passion, time, and creativity in science. An eloquent example is this Special Issue, in which seven female-led groups working in the biosensors field addressed important issues, presenting original and inspiring solutions. Seven contributions are collected in this Special Issue, describing sensors for the selective detection of biogenic amines for food safety; the detection of glial fibrillary acidic protein and interleukin-6 from sweat for continuous, prognostic monitoring of traumatic brain injuries; the detection of low concentrations of ammonia and nitric oxide by using heterostructures based on cobalt phthalocyanines and gold nanoparticles; the non-enzymatic detection of glucose using copper–nickel electrodes; and the detection of trimethylamine N-oxide using TMAO reductase, glucose oxidase, and catalase immobilized on an electrode surface. Two interesting reviews focus on the use of surface plasmon resonance (SPR), localized SPR (LSPR), and fiber-optic SPR (FO-SPR) systems for aquaculture, and several in vitro biosensing schemes with different transductions (piezoelectrical, electrochemical, and optical) based on cysteamine/glutaraldehyde chemistry for the immobilization of specific human biomarkers from body fluids are discussed.

The potential detection of biogenic amines (BAs) in real time as an indicator of food freshness and quality via the use of smart packaging is important to ensure food safety and to fulfil consumer demands. To this end, colorimetric sensors are considered as one of the most feasible solutions. In the manuscript submitted by E. Michelini and co-workers [1], a user-friendly colorimetric sensing paper able to detect BAs via the naked eye is described. The sensing molecule is aglycone genipin, a natural cross-linking agent extracted from gardenia fruit, which is able to bind BAs, producing water-soluble blue pigments. The paper sensor was applied in chicken meat quality monitoring and a quantitative analysis was performed with image acquisition via a smartphone camera, achieving a limit of detection equivalent to 0.1 mM of putrescine. The future use of this sensor was envisaged after integrating the sensor into smart packaging and analyzing commercial chicken meat samples stored at different temperatures; the results confirmed the potential applicability of the sensor as a smart label. In conclusion, the proposed sensor could represent a facile tool for the food packaging industry in designing innovative smart packaging for the benefits of suppliers, retailers, and consumers [1].

Non-invasive approaches for obtaining important biochemical information are detailed in the manuscript submitted by Dr. Shalini Prasad’s team [2]. The team demonstrated the use of a noninvasive, sweat-based, dual biomarker sensor by continuous monitoring of glial fibrillary acidic protein (GFAP) and interleukin-6 (IL-6) in a human sweat analog and in human sweat. To measure the sensor response, electrochemical impedance spectroscopy (EIS) and chronoamperometry (CA) were used. Using EIS, the SWEATSENSER was able to detect GFAP and IL-6 in the sweat analog with an estimated limit of detection (LOD) for GFAP detection of 14 pg mL^−1^ and for IL-6 detection of 10 pg mL^−1^. The sensor demonstrated the ability to detect the relative reactivities of GFAP and IL-6 in simulated profiles of mild and moderate TBIs in human sweat [2].

New heterostructures based on cobalt phthalocyanine (CoPc) films decorated with gold nanoparticles (AuNPs) by gas phase methods and drop-casting (DC) were designed and their chemiresistive sensor responses to low concentrations of NO (10–50 ppb) and NH_3_ (1–10 ppm) were investigated by the team headed by Tamara Basova [3]. The composition, structure, and morphology of the resulting hybrid films were studied by X-ray photoelectron spectroscopy and inductively coupled plasma atomic emission (ICP-AES) spectroscopy, as well as electron microscopy methods, to determine the effects of these properties on the hybrid film sensor response to ammonia and nitric oxide. The detection limits of the Au/CoPc heterostructure with a gold content of ca. 2.1 µg cm^−2^ for NH_3_ and NO were 0.1 ppm and 4 ppb, respectively. It was shown that Au/CoPc heterostructures can be used for the detection of NH_3_ in a gas mixture simulating exhaled air (N_2_—74%, O_2_—16%, H_2_O—6%, and CO_2_—4%) [3].

The non-enzymatic electrochemical detection of glucose in near-neutral solutions based on copper–nickel electrodes, which were electrochemically modified in phosphate-buffered saline, was proposed by the team headed by Aida Ebrahimi. Nickel and copper were deposited using chronopotentiometry, followed by a two-step annealing process in air and electrochemical stabilization in PBS. The morphology and chemical composition of the electrodes were characterized using scanning electron microscopy and energy-dispersive X-ray spectroscopy. Cyclic voltammetry was used to measure the oxidation reaction of glucose in sodium sulfate on the PBS–Cu–Ni working electrodes, enabling the detection of glucose with an LOD of 4.2 nM, a dynamic response of 5 nM–20 mM, and a sensitivity of 5.47 ± 0.45 μA cm^−2^/log10 (mole L^−1^) at an applied potential of 0.2 V [4].

In another interesting approach, the team led by Ulla Wollenberger described an amperometric trimethylamine N-oxide (TMAO) biosensor, where TMAO reductase (TorA), glucose oxidase (GOD), and catalase (Cat) were immobilized on the electrode surface, enabling measurements of mediated enzymatic TMAO reduction at low potential under ambient air conditions [5]. The oxygen anti-interference membrane composed of GOD, Cat, and polyvinyl alcohol (PVA) hydrogel, together with the glucose concentration, was optimized until the O_2_ reduction current of the Clark-type electrode was completely suppressed for at least 3 h. The TMAO sensor operates at a potential of −0.8 V vs. Ag/AgCl (1 M KCl), where the reduction of methylviologen (MV) is recorded. The sensor signal depends linearly on TMAO concentration (between 2 µM and 15 mM), with a sensitivity of 2.75 ± 1.7 µA mM^−1^. The developed biosensor is characterized by a response time of about 33 s and an operational stability of over 3 weeks. Furthermore, measurements of TMAO concentrations were performed in 10% human serum, where the lowest detectable concentration was determined to be 10 µM TMAO [5].

The aquaculture sector has faced severe challenges due to significant cases of environmental pollution, pathogen outbreaks, and the lack of traceability, compromising the quality of its products. Such a context has prompted many researchers to work on the development of novel, affordable, and reliable technologies, many based on nanophotonic sensing methodologies. The review written by the team of Melissa Marlene Rodriguez-Delgado regarding emerging technologies, such as surface plasmon resonance, localized SPR, and fiber-optic SPR systems, showed that all these technologies overcome many of the drawbacks of conventional analytical tools in terms of portability, reagent and solvent use, and the simplicity of sample pre-treatments, which would lead to a more sustainable and profitable aquaculture [6]. In their review, a variety of research detailing the recent advances in these emerging methodologies that can be used to comprehensively monitor the various operations at different commercial stages of farmed aquaculture is presented, as well as the challenges and prospects of developing plasmonic-based sensors for aquaculture [6].

The second review in this Special Issue focuses on the development of biosensors used to confirm the absence/presence of an abnormal level of specific human biomarkers. In this context, for the first time, the review by Dr. Rodica Ionescu summarizes published work over the past 10 years on the pre-functionalization of standard and nanostructured solid/flexible supports with cysteamine and glutaraldehyde chemicals for robust protein immobilization and detection of biomarkers in body fluids (e.g., serum, saliva, and urine) using three transductions (piezoelectrical, electrochemical, and optical) [7].

As a conclusion of this Special Issue, I would like to underline that the role of Special Issues devoted to female scientists is to encourage women in science to believe in their strengths and to follow their passions. Women are ambitious and tenacious, they have incredible resilience and communication skills, and can face all the challenges related to the current environment. This Special Issue proves that women scientists can address a large variety of subjects of interest in the field of biosensors.
